# The identification of carbon dioxide mediated protein post-translational modifications

**DOI:** 10.1038/s41467-018-05475-z

**Published:** 2018-08-06

**Authors:** Victoria L. Linthwaite, Joanna M. Janus, Adrian P. Brown, David Wong-Pascua, AnnMarie C. O’Donoghue, Andrew Porter, Achim Treumann, David R. W. Hodgson, Martin J. Cann

**Affiliations:** 1 Department of Biosciences, Durham University, South Road, Durham, DH1 3LE UK; 2 Biophysical Sciences Institute, Durham University, South Road, Durham, DH1 3LE UK; 3 Department of Chemistry, Durham University, South Road, Durham, DH1 3LE UK; 4 Centre for Sustainable Chemical Processes, Durham University, South Road, Durham, DH1 3LE UK; 5 NUPPA, The Protein Facility, Newcastle University, Cookson Building, Newcastle upon Tyne, NE2 4HH UK

## Abstract

Carbon dioxide is vital to the chemistry of life processes including metabolism, cellular homoeostasis, and pathogenesis. CO_2_ is generally unreactive but can combine with neutral amines to form carbamates on proteins under physiological conditions. The most widely known examples of this are CO_2_ regulation of ribulose 1,5-bisphosphate carboxylase/oxygenase and haemoglobin. However, the systematic identification of CO_2_-binding sites on proteins formed through carbamylation has not been possible due to the ready reversibility of carbamate formation. Here we demonstrate a methodology to identify protein carbamates using triethyloxonium tetrafluoroborate to covalently trap CO_2_, allowing for downstream proteomic analysis. This report describes the systematic identification of carbamates in a physiologically relevant environment. We demonstrate the identification of carbamylated proteins and the general principle that CO_2_ can impact protein biochemistry through carbamate formation. The ability to identify protein carbamates will significantly advance our understanding of cellular CO_2_ interactions.

## Abstract (web summary)

Carbon dioxide can interact with proteins to form carbamate post-translational modifications. Here, the authors developed a strategy to identify carbamate post-translational modifications by trapping carbon dioxide and subsequently identifying the carbamylated proteins.

## Introduction

Protein functionalities can be extended and modulated by enzyme-catalysed and spontaneous post-translational modifications (PTMs)^1^ such as phosphorylation, nitrosylation, acetylation, methylation, hydroxylation, glycosylation and the attachment of other small proteins. The earliest known PTM, the addition of CO_2_ to protein amino groups, was uncovered in two classic studies of early physiology. Bohr and co-workers demonstrated that the haemoglobin oxygen saturation curve was responsive to the partial pressure of CO_2_ while Christiansen and co-workers showed that CO_2_ uptake by the blood at constant *p*CO_2_ was increased by the presence of O_2_^2,3^. Henriques then used kinetic evidence to postulate the direct combination of CO_2_ with the free amino groups on haemoglobin^4^ and Ferguson and Roughton confirmed this through direct chemical analyses^5^. The site of CO_2_ binding was demonstrated^6,7^ to occur at the Val-1β site^8^ linked to the O_2_ binding state of the β-chain^9^. Physiologically, the reaction between the α-amino group and CO_2_ stabilises the deoxygenated form of the protein^10^. Reaffirmation of the role of protein carbamylation is observed in Ribulose-1,5-bisphosphate carboxylase/oxygenase (RuBisCO) which fixes atmospheric CO_2_ in plants using Mg^2+^ and inorganic carbon as co-factors^11^. Experiments with RuBisCO demonstrated that CO_2_ is acting not only as a substrate for the carboxylase reaction, but also as a co-factor that binds to an alternative site on the enzyme^12–14^. Fixation of ^14^CO_2_ to RuBisCO in complex with Mg^2+^ and a carboxyarabinitol bisphosphate carboxylase reaction intermediate confirmed CO_2_-mediated carbamylation of an *ε*-amino group of lysine within the active site^14,15^.

**Fig. 1:**
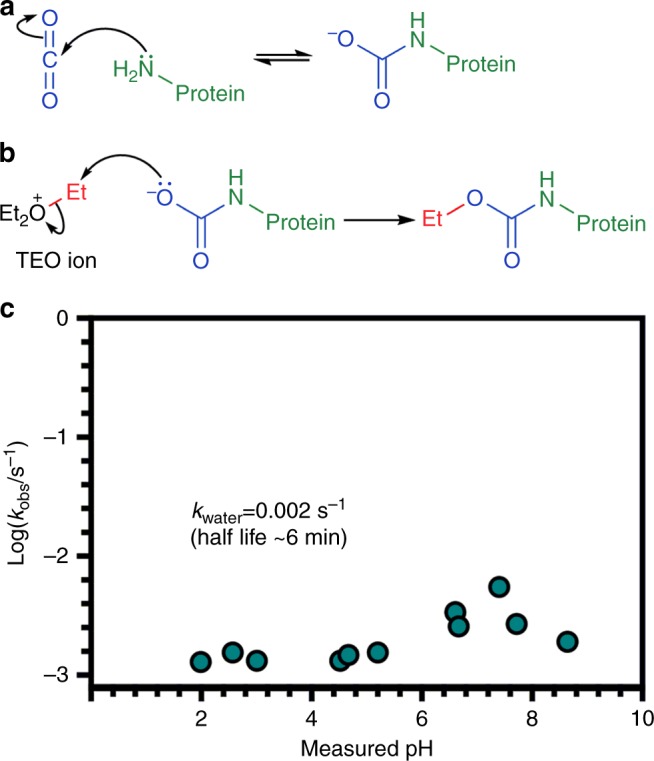
Protein carbamate formation and trapping with TEO. **a** Carbamates form through the reversible reaction between CO_2_ and neutral amine groups. **b** Proposed trapping mechanism of a protein carbamate with TEO. TEO transfers an ethyl group (red) to the anionic carbamate derived from CO_2_ (blue) and protein primary amine (green). **c** Observed pseudo first-order rate constants, *k*_obs_, for the hydrolysis of TEO plotted as a function of pH (where *k*_obs_ = *k*_water_ + [*k*_hydroxide._*K*_W_/10^–pH^])

The work of Lorimer and co-workers provided a mechanism for carbamate formation whereby nucleophilic attack of a neutral amine on CO_2_ converts the amine to an anionic group with the possibility for modulating protein activity^14^ (Fig. 1a). Thus the identification of carbamylation as a PTM in haemoglobin and RuBisCO led to the proposal that carbamylation of neutral *N*-terminal *α*-amino groups and the *ε*-amino group of lysine side chains could form the basis of a widespread mechanism for biological regulation^16^.

The carbamate modification is readily reversible but can be maintained by the protein environment, either through stabilising interactions such as is the case for RuBisCO or through a privileged pH environment such as within haemoglobin. Evidence for the frequency of carbamate formation on protein has been provided by computation that predicts as many as 1.3% of large proteins could bind CO_2_ by carbamylation^17^. In support of a general biological relevance for such a mechanism of CO_2_-binding in protein, several carbamates have been identified on lysine side chains in crystal structures including urease^18^, alanine racemase^19^, transcarboxylase 5 S^20^, class D β-lactamase^21^, and phosphotriesterase^22^. A recent carbamate was proposed in the Cx26 connexin hemi-channel, which opens in response to CO_2_ in respiratory control and releases ATP^23^. Here, carbamate formation on Lys125 is hypothesised to stabilise an electrostatic interaction with Arg104 in the neighbouring subunit of the hexamer thus constraining the hemi-channel in the open state in response to high [CO_2_]. The lability of such exchangeable carbamates outside of the cellular environment makes the identification of this PTM a significant analytical challenge. Soft ionisation mass spectrometry techniques have had limited success in demonstrating the presence of carbamate PTMs but were unable to identify locations of modification^24^.

Here we describe the development and use of a methodology for the trapping of carbamates under physiologically relevant conditions. The development of the carbamate-trapping method was established on amino acids, peptides, and proteins. The results demonstrate that labile carbamates can be selectively derivatised by ethylation thus stabilising them for downstream analysis. We demonstrate that the approach can identify the known carbamate on haemoglobin and go on to apply the method to the proteome of a model organism to identify CO_2_-binding sites. We demonstrate the general principle that the activity of an identified protein is altered in response to CO_2_ on mutation of the identified CO_2_-binding site.

Our results lead us to propose that CO_2_-mediated protein binding at exchangeable sites exists beyond haemoglobin. This may have profound consequences for our understanding of cellular responses to CO_2_.

## Results

### Triethyloxonium ion-mediated carbamate trapping on amino acids and peptides

The diazomethane derivative trimethylsilyl-diazomethane (TMS-DAM) has been previously used to alkylate carbamates in CO_2_-bubbled solutions containing amines^25^; however, TMS-DAM is not water soluble so it is unsuitable for work with proteins. We therefore investigated a water-soluble reagent to alkylate and therefore trap labile CO_2_ PTMs. Meerwein salts have been used to modify carboxylic groups on proteins through *O-*methylation^26^. Limited kinetic data on the hydrolysis of the Meerwein reagent triethyloxonium (TEO) suggested a half-life of ~7 min in aqueous solution, and so we explored this system in more detail with a view to developing the reagent for trapping labile carbamates on protein (Fig. 1b). We used an indicating buffer method^27^ to determine the pH-hydrolysis characteristics of TEO for 2 < pH < 9 and observed *k*_water_ = 2.23 × 10^−3^±1.22 × 10^−3^ s^–1^ (S.D), corresponding to a *t*_½_ of approximately 6 min at physiological pH (Fig. 1c, Supplementary Figure 1–2, Supplementary Table 1–2, Supplementary Methods). This rate of hydrolysis allows carbamate trapping and pH control of the experiment to take place on a convenient laboratory timescale.

**Fig. 2:**
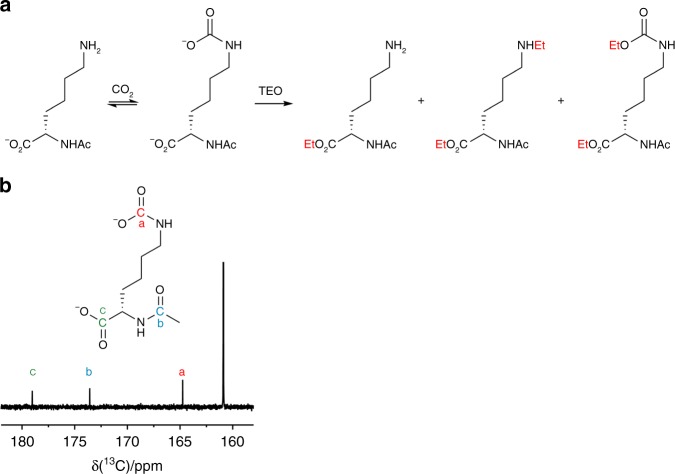
Carbamate trapping on the *ε*-NH_2_ group of *α*-*N*-acetyl-lysine. **a** *N*-acetyl-lysine-carbamate formation and trapping (*O*-ethylation) by TEO. **b** ^13^C-NMR spectrum demonstrating the formation of a carbamate on *N*-acetyl-lysine by the appearance of a peak at 164 ppm

**Fig. 3:**
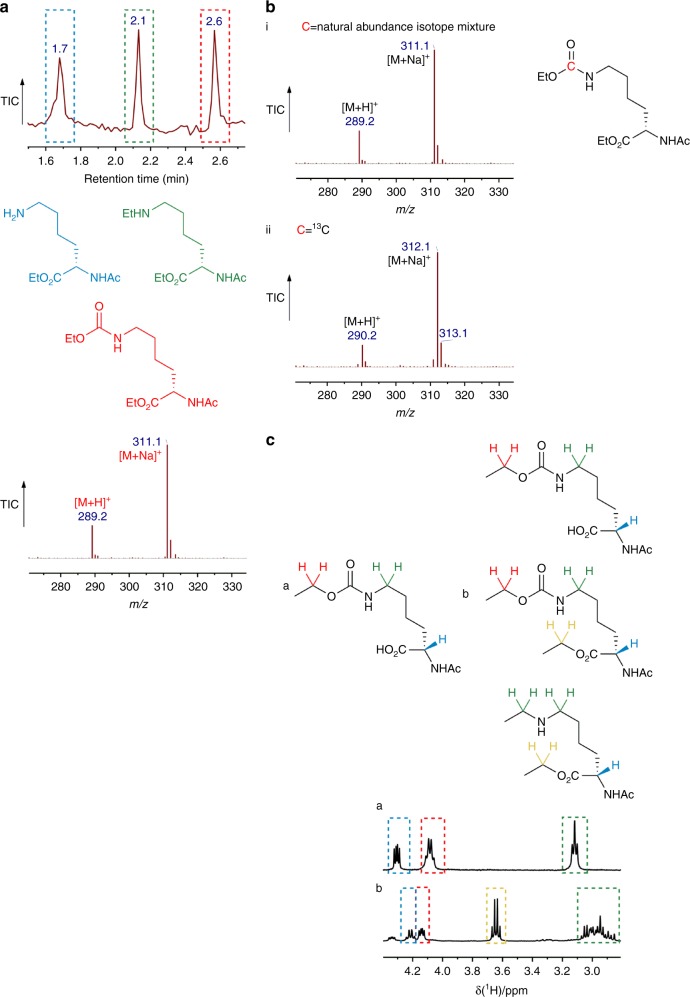
Characterisation of carbamate trapping on the *ε*-NH_2_ group of *α*-*N*-acetyl-lysine. **a** Total ion chromatogram of the carbamate-trapping reaction mixture of *α*-*N*-acetyl-lysine with TEO, and *m/z* profile for species at retention time ~2.6 min. The major products of the trapping reaction are *N*-acetyl-lysine ethylated on the *α*-carboxylate group (retention time ~1.7 min), *α*-*N*-acetyl-lysine ethylated on the *α*-carboxylate and *ε*-NH_2_ groups (retention time ~2.1 min) and *α*-*N*-acetyl-lysine ethylated on the *ε*-carbamate and the *α*-carboxylate groups (retention time ~2.6 min). **b** MS trace demonstrating the increase of one mass unit from ^12^C (i) with the use of ^13^C labelled CO_2_ (ii). **c**. ^1^H-NMR spectra comparing ethyloxycarbonyl signals between **a** chemically synthesised *α*-*N*-acetyl-*ε*-*N*-ethyloxycarbonyl-lysine and **b** ethylation products formed during the trapping experiments between *α*-*N*-acetyl-lysine, CO_2_ and TEO (key CH_2_ signals highlighted in red)

We sequentially validated the potential of TEO as a carbamate-trapping agent on amino acid, peptide and protein substrates. Experiments with amino acid and peptide substrates were performed at pH 8.5 to promote carbamate formation in these systems. Initial investigation centred on whether TEO could trap a carbamate on *α*-*N*-acetyl-lysine under aqueous conditions. We hypothesised that a carbamate would form on the ɛ-amino group that could be subsequently trapped with TEO (Fig. 2a). A solution of *α*-*N*-acetyl-lysine was incubated with excess ^13^CO_2_/H^13^CO_3_^−^ at pH 8.5 and the formation of a carbamate was confirmed by the presence of a peak at 164 ppm by ^13^C NMR spectroscopy (Fig. 2b)^28^. Separately, TEO was added to an *α*-*N*-acetyl-lysine/CO_2_/HCO_3_^−^ mixture at constant pH and the reaction products were analysed by LC-ESI-MS. The ethylation mixture was resolved into three major components that demonstrated the trapping of ε-carbamate was successful with side products including *C*-terminal and *N*-ethylation (Fig. 3a). To further confirm the formation of the carbamate, the buffered NaHCO_3_ solution used to provide CO_2_ was replaced with NaH^13^CO_3_, which resulted in the expected 1 Da *m/z* increase on MS analysis (Fig. 3b). The ethylation product mixture was extracted into ether and its ^1^H NMR spectrum (Figure 3cii) was compared to a chemically synthesised standard *ε*-ethyloxycarbonyl-lysine (Figure 3ci). The product spectrum shows key signals consistent with *N-*carboxyethylation at *δ*~4.25 ppm and ~1.2  ppm that corroborate the findings from LC-ESI-MS (Fig. 3a).

**Fig. 4:**
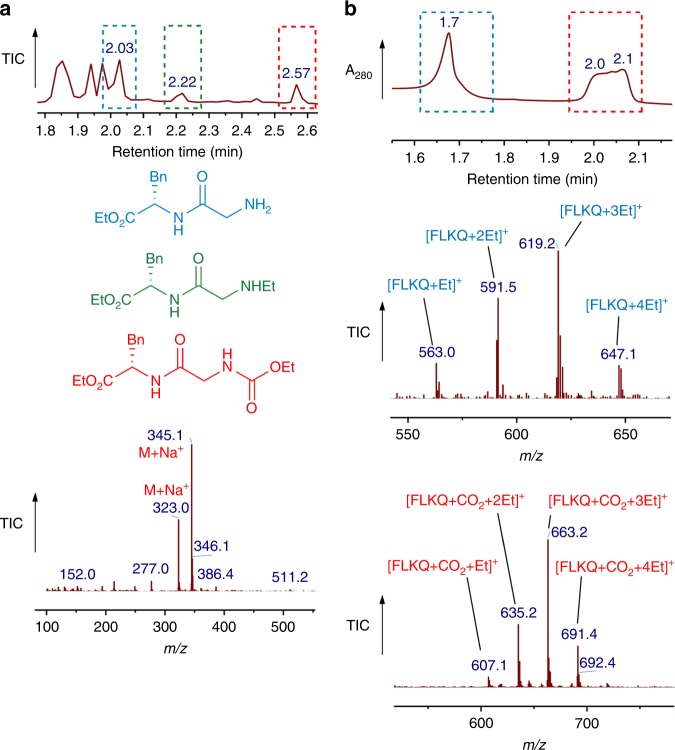
Carbamate trapping on di- and tetrapeptides. **a** Total ion chromatogram of the carbamate-trapping reaction mixture of Gly-Phe with TEO, and *m/z* profile for species at retention time 2.57 min. The major products of the trapping reaction are Gly-Phe ethylated on the Phe-*α*-carboxylate (retention time 2.03 min), Gly-Phe ethylated on the *α*-carboxylate of Phe and the *α*-NH_2_ group of Gly (retention time 2.22 min) and Phe-Gly ethylated on the carbamate of the *α*-NH_2_-group of Gly and the *α*-carboxylate group of Phe (retention time 2.57 min). **b** Total ion chromatogram of the carbamate-trapping reaction mixture of FLKQ tetrapeptide with TEO and *m/z* profiles for species at retention time 1.7 and 2.0–2.1 min. The major products of the trapping reaction are shown as FLKQ with 1-4 ethylation groups (retention time 1.7 min) and FLKQ with 1 trapped carbamate and 1-4 ethylation groups (retention time 2.0–2.1 min)

After confirming carbamate trapping on *α*-*N-*acetyl-lysine we examined peptide systems, focusing first on the dipeptide Gly-Phe. A CO_2_-TEO trapping reaction of Gly-Phe at pH 8.5 was investigated by LC-ESI-MS and confirmed successful carbamate trapping on the *N*-terminus of the Gly-Phe dipeptide (Fig. 4a) alongside *C*-terminal *O*-ethylation and *N*-terminal ethylation side products. Carbamate trapping on tetrapeptide FLKQ was then investigated by LC-ESI-MS (Fig. 4b), yielding a trapped carbamate on either the *N*-terminus or the lysine side chain. Together, these data demonstrate that it is possible to form carbamates on the α- and ε-amino groups of peptides under physiologically relevant aqueous conditions and trap them by *O*-ethylation with TEO.

### Triethyloxonium ion-mediated carbamate trapping on protein

Having demonstrated that TEO is a suitable tool to trap carbamates on amines, we sought to use it for the discovery of protein carbamates that would represent sites for CO_2_ binding that are exchangeable with the environment. We hypothesised that selective CO_2_ binding to protein through carbamate formation would occur in structurally privileged sites that have evolved to facilitate carbamate formation. For example, CO_2_ binding to haemoglobin at the Val-1β site occurs through such a privileged environment. The formation of non-specific carbamates at other sites on protein is proportionately much less likely due to the p*K*_a_ for the Lys ɛ-amino group being ~9–10.

**Fig. 5:**
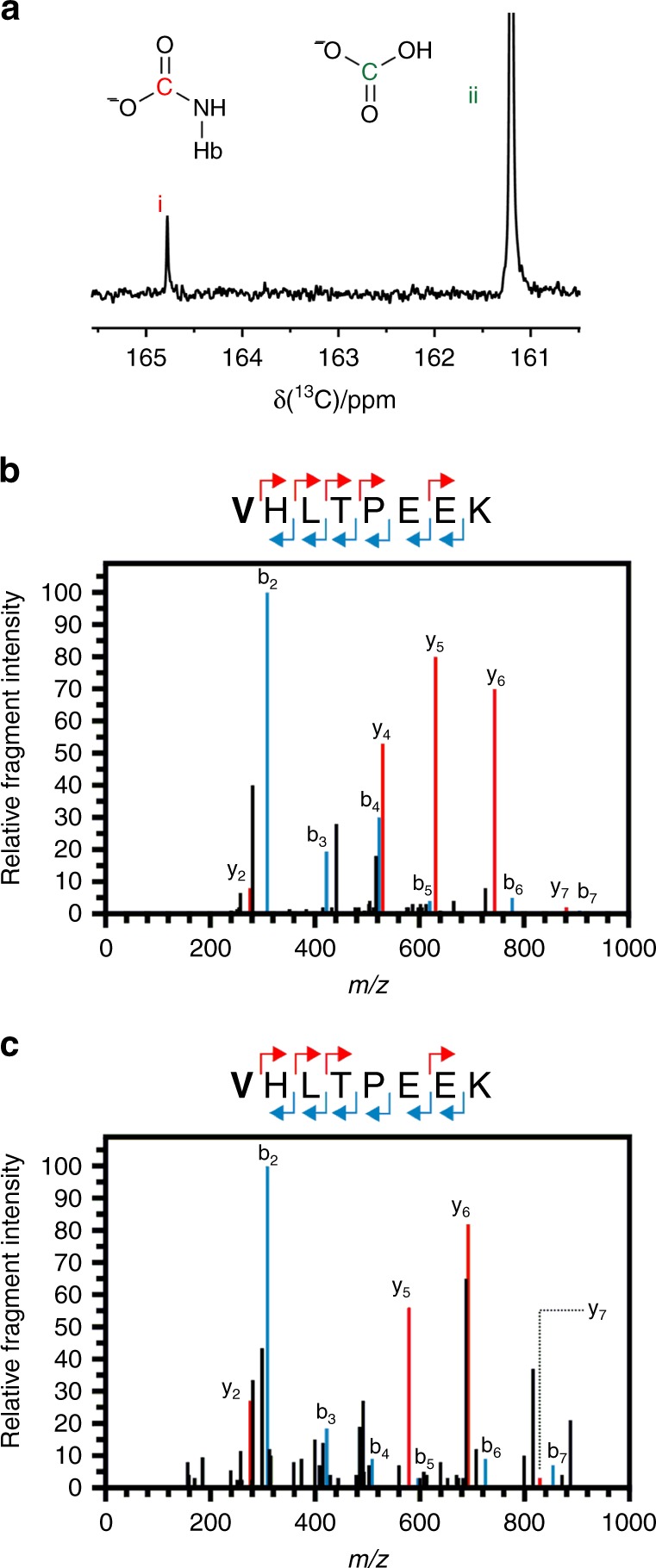
Identification of the exchangeable CO_2_-binding site on haemoglobin. **a** ^13^C-NMR spectrum demonstrating the formation of a carbamate on haemoglobin by the appearance of a peak at 164 ppm (i) together with a peak from H^13^CO_3_^−^ in solution (ii). **b** A plot of relative fragment intensity versus mass/charge ratio (*m/z*) for fragmentation data from MS-MS identifying an ethyl-trapped carbamate on the *N-*terminal valine of the haemoglobin β-chain. The experiment used purified haemoglobin. The peptide sequence above indicates the identification of predominant ^+1^y (red) ^+1^b (blue) ions by MS-MS shown in the plot. The modified residue is indicated in bold. The experiment also identifies a further ethylation on E7. **c** A plot of relative fragment intensity versus mass/charge ratio (*m/z*) for fragmentation data from MS-MS identifying an ethyl-trapped carbamate on the *N-*terminal valine of the haemoglobin β-chain. The experiment used whole red blood cells. The peptide sequence above indicates the identification of predominant ^+1^y (red) ^+1^b (blue) ions by MS-MS shown in the plot. The modified residue is indicated in bold

The previous experiments with amino acids and peptides had been performed at pH 8.5 to promote carbamate formation by driving the ɛ-amino group protonation equilibrium towards the uncharged state. However, experiments with protein were performed at pH 7.4 to replicate a cellular environment which does not enhance carbamate formation. Carbamates will therefore only form in privileged environments. We first investigated TEO-mediated CO_2_ trapping on the *N-*terminal valine of the haemoglobin β-chain. Carbamate formation was confirmed using ^13^C-NMR spectroscopy by the observation of a signal at 164 ppm which matched literature values^29^ (Fig. 5a).

We trapped CO_2_ onto human haemoglobin with TEO and analysed the trypsin-digested products by ESI-MS. A carbamate was identified on the α-amine of the β-chain *N*-terminal valine (peptide mass 1024.10 Da), consistent with the literature (Fig. 5b)^8^. Peptides carrying a carbamate were identified both with and without ethylation on E7. Ethylation of alternative sites therefore does not influence the ability of the method to trap carbamates. The experiment was repeated after 4% SDS addition and removal to denature haemoglobin and thus destroy the local privileged environment required for carbamate formation. No trapped carbamate was observed in an experiment performed under these conditions. Carbamate formation therefore requires a structure-dependent privileged environment within the protein. Removal of the protein structure by SDS destroyed this privileged environment and thus the carbamate could not form. The trapping methodology is therefore able to identify known functional carbamates on proteins under physiologically relevant conditions of pH and [CO_2_].

We trapped CO_2_ onto intact rabbit red blood cells with TEO to confirm that the methodology is able to identify carbamates in the normal cellular environment. Trypsin-digested whole cells were analysed by LC-ESI-MS. The expected carbamate on haemoglobin was again identified on the α-amine of the β-chain *N*-terminal valine (Fig. 5c). This experiment demonstrates that the developed methodology can also be applied within a cell and that the results obtained are identical to those from isolated protein.

### The identification of protein carbamates

**Fig. 6:**
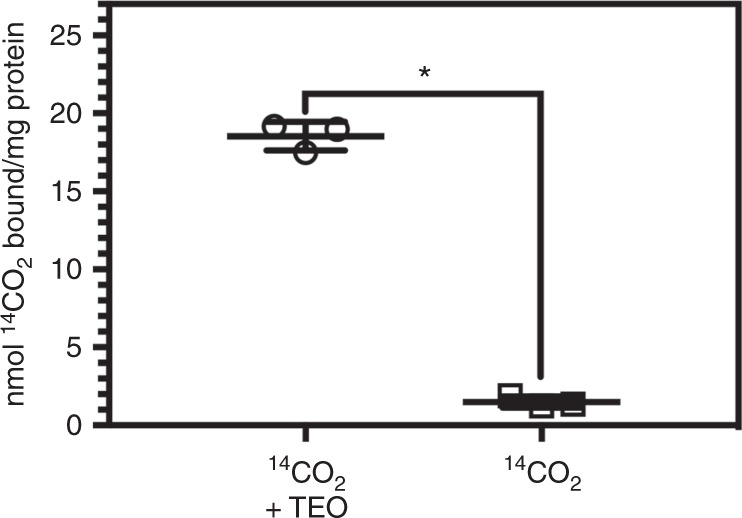
Identification of exchangeable CO_2_-binding site on *Arabidopsis* protein extract. ^14^CO_2_ trapped onto protein extract of *Arabidopsis thaliana* (*p < 0.0001, two-tailed *t*-test, *n* = 3 independent replicates, *t* = 29.85, d*f* = 4, ±S.E.M.)

We hypothesised that the TEO trapping methodology could be used to isolate previously unidentified CO_2_-binding sites on proteins. We therefore performed a small-scale screen of the proteome of a model organism to establish the general principle that CO_2_ can form labile interactions with protein through carbamate formation. We selected the CO_2_-fixing organism *Arabidopsis thaliana* for study as we hypothesised it would be most likely to utilise protein carbamylation as a mechanism to couple CO_2_ availability to protein function. Extracts of soluble proteins derived from the leaves of *A. thaliana* were incubated with NaH^14^CO_3_ and subjected to TEO-trapping (Fig. 6). Little ^14^CO_2_ was incorporated into the protein extracts in the absence of TEO. The inability to identify protein-bound ^14^CO_2_ in the absence of TEO was due to the ready reversibility of carbamylation that leads to degassing of the sample during preparation for analysis. The trapped proteome contained significant levels of ^14^C, even when accounting for 50% of the total protein sample being Rubisco. We concluded that *Arabidopsis* protein extract contains CO_2_-interacting proteins carbamylated at labile sites exchangeable with the environment. We therefore proceeded to identify a subset of these carbamylated proteins.

To identify carbamylated proteins within *Arabidopsis*, soluble leaf protein was equilibrated with CO_2_/HCO_3_^−^ at pH 7.4 and TEO was added. The trapping reaction mixture was digested with trypsin and samples were analysed by LC-MS-MS. The data were interrogated for variable post-translation modifications on lysine with masses of 72.0211 Da (trapped carbamate) and 28.0313 Da (*O*-ethylation on glutamate and aspartate side chains). Occasional *N*-ethylation of the lysine or arginine amino group was also observed (as seen in Fig. 2a, for example). Carbamate formation occurs before TEO addition and *N*-ethylation of the lysine amino group can only occur on an unmodified lysine so therefore does not compete with carbamate trapping.

**Fig. 7:**
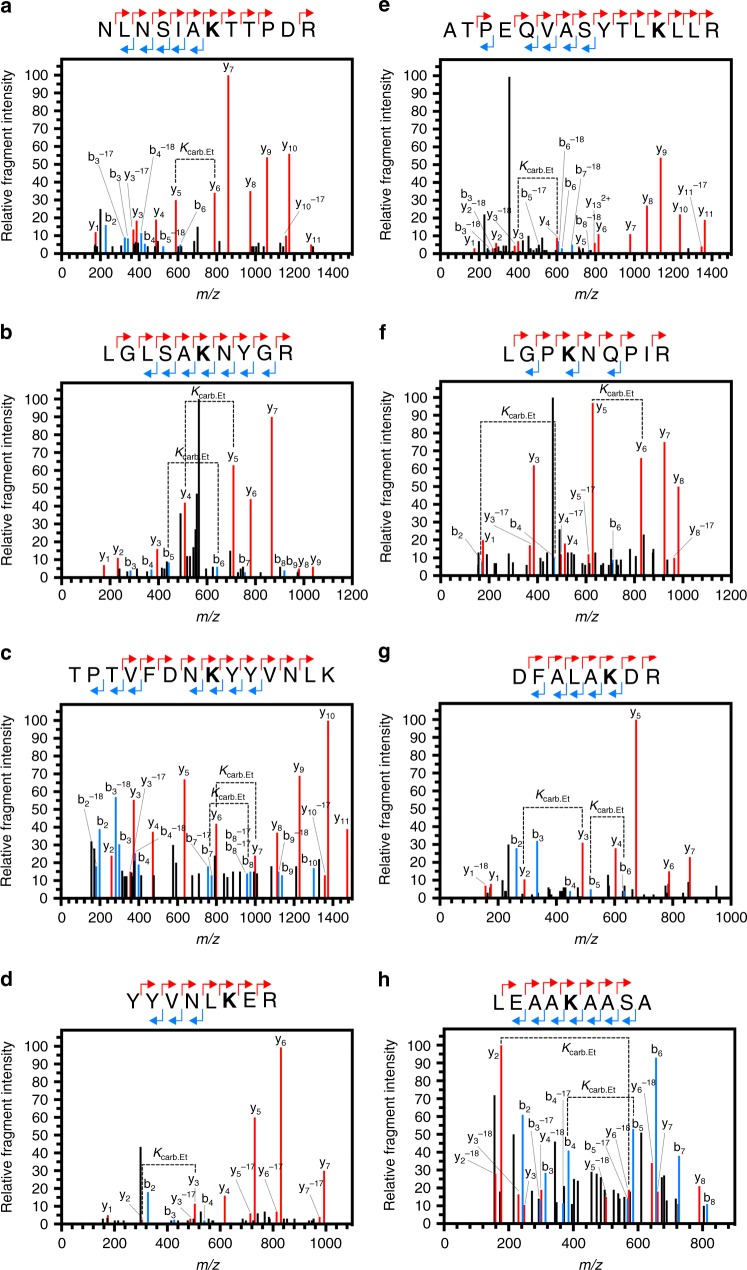
The identification of CO_2_-binding proteins. MSMS spectra of peptides that were identified with ethyl-trapped carbamates on Lys residues. Panel **b** is a CID spectrum acquired on an LTQ Orbitrap XL mass spectrometer (low resolution), panel **e** was acquired on a QStar Pulsar mass spectrometer QqTOF with intermediate resolution and panels **a**, **c**, **d**, **f**–**h** are CID spectra acquired on a high resolution QqTOF mass spectrometer (Sciex TT6600). The peptide sequences above each panel indicate the assignment of predominantly singly charged y (red) and b (blue) ions. The modified residue is indicated in bold. *K*_carb.Et_ indicates the molecular weight difference between ions diagnostic of the modified lysine. **a** Lysine 65 of At2g38540. **b** Lysine 183 of AtCG00490. **c** Lysine 262 of At3g49120. **d** Lysine 268 of At3g49120. **e** Lysine 293 of At2g21330. **f** Lysine 251 of At3g54400. **g** Lysine 109 of At4g21280. **h** Lysine 208 of At4g25100

Two additional criteria were used to support the identification of carbamylated proteins and eliminate false positives. First, modified residues were discarded unless at least two y-ions and two b-ions confirmed the location of the PTM under MS–MS conditions. Second, only peptides that contained an internal lysine residue (missed cleavage) were accepted because carbamylation removes the positive charge on the lysine that is essential for cleavage site recognition by trypsin. This is analogous to the removal of tryptic cleavage sites through lysine acetylation^31^. We identified eight CO_2_ binding sites in *Arabidopsis* (Table 1, Fig. 7). Assignment of the MSMS spectra was manually verified and supported by high mass accuracy measurements of the fragment ions in 7 out of the 8 spectra (Supplementary Data 1). Together these data suggest that trapping CO_2_ with TEO can be used for the discovery of proteins post-translationally modified by CO_2_.

**Table 1:** Carbamylated proteins in *A. thaliana*

Genome Identification Number	Protein	Residue
At2g38540	Lipid-transfer protein	K65
AtCG00490	Rubisco Large Chain	K183
At3g49120	Peroxidase	K262 K268
At2g21330	FBA1	K293
At3g54400	Eukaryotic aspartyl protease family protein	K251
At4g21280	PSBQA	K109
At4g25100	Fe Superoxide dismutase 1	K208

### Validation of a protein carbamate

**Fig. 8:**
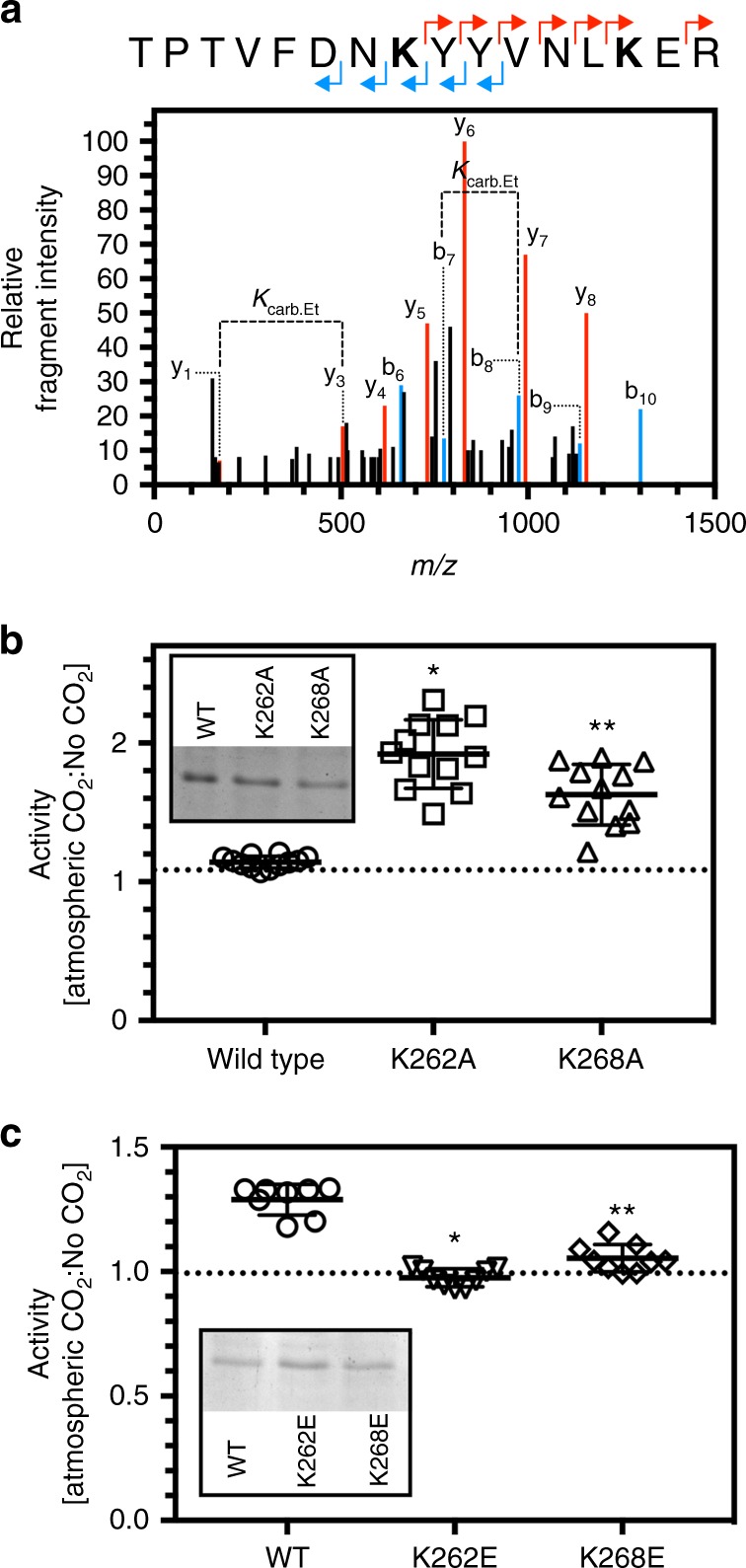
Biochemical validation of *At*PRX34 as a carbamylated protein. **a** MSMS spectra of peptides containing ethyl-trapped carbamates on Lys 262 and Lys 268 of At3g49120. The peptide sequences above the panel indicate the assignment of predominantly singly charged y (red) and b (blue) ions. The modified residue is indicated in bold. *K*_carb.Et_ indicates the molecular weight difference between ions diagnostic of the modified Lys. **b** The ratios of the specific activities of wild type, K262A or K268A *At*PRX34 protein at atmospheric CO_2_ or in the absence of CO_2_ (* *p* < 0.0001 compared to wild type, one-way ANOVA with Dunn Bonferroni multiple comparison test, *n* = 12 independent replicates, Kruskal–Wallis statistic = 26.4, ±S.D.; ** *p* < 0.0028 compared to wild type, one-way ANOVA with Dunn Bonferroni multiple comparison test, *n* = 12 independent replicates, Kruskal-Wallis statistic = 26.4, ±S.D.). Inset—purification of recombinant *At*PRX34 proteins (1.0 μg; SDS/PAGE analysis and Coomassie Blue staining). **c** The ratios of the specific activities of wild type, K262E or K268E *At*PRX34 protein at atmospheric CO_2_ or in the absence of CO_2_ (* *p* < 0.0001 compared to wild type, one-way ANOVA with Dunn Bonferroni multiple comparison test, *n* = 9 independent replicates, Kruskal–Wallis statistic = 19.53, ±S.D.; ** *p* < 0.0194 compared to wild type, one-way ANOVA with Dunn Bonferroni multiple comparison test, *n* = 9 independent replicates, Kruskal–Wallis statistic = 19.53, ±S.D.). Inset- Purification of recombinant PRX34 proteins (1.0 μg; SDS/PAGE analysis and Coomassie Blue staining)

We hypothesised that CO_2_ would influence the activity of these discovered proteins at the identified site. To demonstrate this, we selected a hit protein for further investigation. The Class III peroxidase PRX34 (*At*PRX34; At3g49120) was identified as a hit by MS-MS (Fig. 7c, d). Two proximal lysine carbamylation sites were identified (MSMS peptide amino acids 255–268 TPTVFDN**K**YYVNLK, proposed carbamylation on K262; MSMS peptide amino acids 263–270 YYVNL**K**ER, proposed carbamylation on K268). We further identified lysine carbamylation on K262 and K268 simultaneously (MSMS peptide amino acids 255-268 TPTVFDN**K**YYVNL**K**ER, proposed carbamylation on K262 and K268) indicating that PRX34 carbamylation does not necessarily occur exclusively on K262 or K268 (Fig. 8a). *At*PRX34 generates H_2_O_2_ in response to microbe-associated molecular patterns suggesting that *At*PRX34 has a role in basal defence responses in the plant^32^. We over-expressed the mature coding sequence (amino acids 31–353; without transit peptide sequence) of wild type *At*PRX34 and both K262A and K268A single site mutants in *E. coli* as His-tagged fusion proteins. We assayed the *At*PRX34 wild type, *At*PRX34 K262A, and *At*PRX34 K268A proteins by measuring their ability to oxidise 2-methoxyphenol in the presence of H_2_O_2_ under conditions of atmospheric CO_2_ (approximately 12 μM CO_2_) and in the absence of CO_2_. We compared the ratio of the specific activities for each protein in the presence and absence of CO_2_ (Fig. 8b) in reaction mixtures with measured final pH of 7.4. Wild type protein showed no difference in specific activity under conditions of atmospheric CO_2_ compared to no CO_2_. However, both the K262A and K268A mutants demonstrated elevated activity under conditions of atmospheric CO_2_ compared to the absence of CO_2_. This result suggests a control mechanism similar to that found within haemoglobin. Carbamylation of the two haemoglobin Val-1β sites reduced the affinity of haemoglobin for O_2_ at a third site. We hypothesise that carbamate formation at K262 and K268 within *At*PRX34 provide a control system for peroxidase activity in the presence of CO_2_. This reduction in activity is clearly altered when either site is mutated and carbamate formation cannot occur (Fig. 8b). The higher reactivity of the mutants leaves the only explanation to be a role in suppression. We were unable to detect a trapped carbamate at K262 in the K268A mutant protein or a trapped carbamate at K268 in the K262A mutant protein by MSMS. This suggests that the lysine at residue 262 or 268 promote or stabilise carbamate formation at the alternative site as evidenced by the identification of the singly carbamylated sites in the wild type proteins (Fig. 7c–d). Mutation of either lysine to alanine makes it less likely for a carbamate to form at the other site. This is manifested as a loss of sensitivity to CO_2_ in the single mutant proteins. The specific activities of the wild type, K262A and K268A proteins at atmospheric CO_2_ are 0.364±0.027, 0.479±0.12 and 0.500±0.099 μmol 1,2-benzoquinone  mg^−1^ min^−1^. These values demonstrate that the altered response to CO_2_ in the mutant protein is a true activity change and not due to a change in specific activity caused by the mutation. No other carbamates were identified on *At*PRX34 therefore CO_2_ is likely able to interact with *At*PRX34 at another site by an alternative carbamate-independent mechanism as previously observed^33^. Carbamylation at either K262 or K268 therefore mitigates the effects of CO_2_ at a third site which would otherwise activate the enzyme.

We hypothesised that mutation of K262 or K268 to glutamate would represent the local charge state of a carbamate at 100% occupancy. We therefore compared the ratio of the specific activities for PRX34 K262E or K268E to the wild type protein in the presence and absence of CO_2_ (Fig. 8c). As before, wild type protein showed only negligible difference in specific activity under conditions of atmospheric CO_2_ compared to no CO_2_. In addition to this both the K262E and K268E mutants demonstrated no change in activity. However, these values were significantly different from the wild type which highlights the possible variability in occupancy of the carbamate in the wild type protein. This variability is not present in the fully occupied glutamate mutants. The specific activities of the wild type, K262E and K268E proteins at atmospheric CO_2_ are 1.653±0.060, 1.477±0.027 and 0.933±0.020 μmol 1,2-benzoquinone  mg^−1^ min^−1^. Specific activities are different between independent preparations of refolded proteins. The ratio of specific activities (Atmospheric CO_2_:No CO_2_) is independent of absolute specific activity and is comparable across preparations and repeatable across experiments.

The data are consistent with a model in which the two carbamate sites present are cooperative in maintaining protein activity levels. If the ability to carbamylate at one site is removed (K262A, K268A) then the difference of activity with changes in CO_2_ levels significantly increases. In mutants that mimic carbamates at 100% occupancy (K262E, K268E) any change to activity due to changes in CO_2_ level is removed.

*At*PRX34 is closely related to four additional Class III peroxidases encoded in the *Arabidopsis* genome (*At*PRX32, *At*PRX33, *At*PRX37 and *At*PRX38). The carbamylated lysines are conserved in all five peroxidases. A future task, therefore, will be to elucidate the physiological function of the individual peroxidases in *Arabidopsis*, the role of CO_2_ in these physiological processes and the impact of the individually carbamylated residues. We therefore demonstrate that under physiologically relevant conditions protein carbamates can be identified in which CO_2_-binding site influences protein biochemistry in vitro.

## Discussion

It is remarkable that so little is known about how CO_2_ influences the function of the proteome, despite its fundamental importance within the cellular environment. Here we describe the general principle that CO_2_ can reversibly bind protein through carbamate formation. The carbamates identified to date by design (haemoglobin, RuBisCO) or fortuitously as stable modifications in crystal structures (urease^18^, alanine racemase^19^, transcarboxylase 5S^20^, class D β-lactamase^21^, and phosphotriesterase^22^) have clear functional roles. This confirms that carbamate formation is a candidate mechanism for protein activity to be directly responsive to environmental CO_2_. However, the majority of these carbamates were discovered incidentally due to the lack of a tool for their direct investigation. We have presented a route to identify such CO_2_-binding sites and provide evidence that such a site can influence protein biochemistry in a CO_2_-dependent manner.

Our method operates under physiologically relevant conditions and successfully identified the known site of carbamate formation in haemoglobin dependent upon its local privileged environment. Analyses of the proteome of *A. thaliana* demonstrated significant^14^CO_2_ binding to protein dependent upon carbamylation as evidenced by the requirement for TEO to trap CO_2_ on protein. A small-scale proteomics screen identified eight carbamylation sites from 3614 proteins. Several other potential sites were ruled out by the stringent conditions used to eliminate potential false positives. Further developments in chromatography should enable us to increase the coverage of the proteome in such CO_2_-trapped samples.

Our trapping method provides the capability for identifying proteins targeted by CO_2_ in any system, which should in turn enable the construction of models for how cellular functions detect and therefore respond to CO_2._ Protein carbamylation is likely to be more widespread than previously suspected and can represent a mechanism by which CO_2_ availability is coupled to protein function. The challenge for the future is to identify protein targets for CO_2_ and the functional roles of the resulting carbamate. It is highly likely that many, if not all, carbamylation sites will be functionally relevant.

In conclusion, we present a method for the identification of carbamylated proteins at the proteome level and show that this PTM is likely to be of biological significance. This method will allow a significant expansion of our current understanding of protein regulation by CO_2_ and provide information concerning the extent to which CO_2_ interacts with the proteome.

## Methods

### CO_2_ trapping

All CO_2_ trapping experiments were carried out in phosphate buffer (4 mL, 50 mM, pH 7.4). This solution was transferred to a TIM856 Titration Manager (Radiometer Analytical) and incubated at 25 °C with stirring. Triethyloxonium tetrafluoroborate (Et_3_OBF_4_; various amounts; details below) was added stepwise with a constant pH being maintained (pH 7.4) through the slow addition of 1 M NaOH solution via the automatic burette. The reaction mixture was stirred, and the pH maintained, for 1 h after the final Et_3_OBF_4_ addition in order to ensure that all TEO was hydrolysed.

### *N*-acetyl-lysine CO_2_ trapping with TEO

*α-N*-acetyl-lysine (5 mg, 0.03 mmol) was dissolved in phosphate buffer (2 mL, 50 mM, pH 8.5). NaHCO_3_ (1.7 mg, 0.02 mmol) was dissolved in phosphate buffer (1 mL, 50 mM, pH 8.5) and added to the *N*-acetyl-lysine solution. The combined mixture was transferred to the Titration Manager, and Et_3_OBF_4_ (100 mg, 0.53 mmol) was added in three equal portions while the pH of the solution was maintained via the automated addition of NaOH solution (1 M). The mixture was stirred for 1 h after the final Et_3_OBF_4_ addition, then lyophilised and re-dissolved in methanol (1 mg/mL) for MS analysis. The sample was analysed using ESI-MS and the trapped carbamylated *N*-acetyl-lysine product was confirmed. ESI-MS: [M+H^+^] 289.17.

### Synthesis of *α*-*N*-acetyl-*ε*-*N*-ethyloxycarbonyl-lysine

*α-N*-acetyl-lysine (50 mg, 0.25 mmol) and NaHCO_3_ (50 mg, 0.60 mmol) were dissolved in dH_2_O (1 mL). Ethyl chloroformate (27 mg, 0.25 mmol) in THF (3 mL) was added with stirring. The mixture was stirred overnight at room temperature, then the solvents were removed under reduced pressure. The precipitate was dissolved in acidified H_2_O (5 mL, pH 2) and the product was extracted into ether (2 × 5 mL). The ether extracts were dried (MgSO_4_) and the solvent was removed under reduced pressure to afford the *ε*-*N*-ethyloxycarbonyl-product (27.7 mg, 40%) as a white solid. ^1^H NMR (400 MHz, D_2_O) δ/ppm 4.29 (1H, dd, *J* = 9.0, 5.0 Hz *α*-C*H*NH), 4.07 (2H, q, *J* = 7.2 Hz C*H*_2_CH_3_), 3.11 (2H, t, *J* = 6.6 Hz *ε*-C*H*_2_NH), 2.03 (3H, s CH_3_CO), 1.90–1.68 (2H, m C*H*_2_CH), 1.50 (2H, quintet, *J* = 6.8 Hz CH_2_C*H*_2_CH_2_), 1.45-1.32 (2H, m, C*H*_2_CH_2_NH), 1.21 (3H, t, *J* = 7.1 Hz C*H*_3_CH_2_).

### Gly-Phe dipeptide trapping

Gly-Phe (8 mg, 0.04 mmol) was dissolved in phosphate buffer (2 mL, 50 mM, pH 8.5). NaHCO_3_ (1.7 mg, 0.02 mmol) was dissolved in phosphate buffer (1 mL, 50 mM, pH 8.5), added to the dipeptide, and the mixture was transferred to the Titration Manager. A freshly made solution of Et_3_OBF_4_ (280 mg, 1.47 mmol) in dH_2_O (1 mL) was added to the mixture in three portions while the pH was maintained by the automated addition of NaOH solution (1 M). The reaction mixture was stirred for 1 h, lyophilised and re-dissolved in methanol (1 mg/mL) for MS analysis. The sample was analysed using ESI-MS and the trapped carbamate Gly-Phe product was confirmed. ESI-MS: [M+H^+^] 323.01.

### FLKQ tetrapeptide trapping

FLKQ (5 mg, 0.009 mmol) was dissolved in phosphate buffer (2 mL, 50 mM, pH 8.5). NaHCO_3_ (1.7 mg, 0.02 mmol) was dissolved in phosphate buffer (1 mL, 50 mM, pH 8.5), added to the tetrapeptide solution, and the mixture was transferred to the Titration Manager. A freshly made solution of Et_3_OBF_4_ (280 mg, 1.47 mmol) in dH_2_O (1 mL) was added to the mixture in three portions while the pH was maintained by the automated addition of NaOH solution (1 M). The reaction mixture was stirred for 1 h then lyophilised and re-dissolved in methanol (1 mg/mL) for MS analyses. The sample was then analysed using ESI-MS and the trapped carbamate FLKQ was confirmed. ESI-MS: [M+H^+^] 663.16.

### Haemoglobin trapping

Human haemoglobin (Hb) (14.5 mg, 0.23 μmol) was dissolved in phosphate buffer (2 mL, 50 mM, pH 7.4). NaHCO_3_ (1.7 mg, 0.02 mmol) was dissolved in phosphate buffer (1 mL, 50 mM, pH 7.4), added to the protein solution, and the mixture was transferred to the Titration Manager. A freshly made solution of Et_3_OBF_4_ (280 mg, 1.47 mmol) in dH_2_O (1 mL) was added to the mixture in three portions while the pH was maintained by the automated addition of NaOH solution (1 M). The reaction mixture was stirred for 1 h then dialysed against dH_2_O (1 L) overnight. The sample was then centrifuged, an aliquot (100 μL) was taken from the supernatant and digested using trypsin. ESI-MS data confirmed a trapped carbamate on the *N*-terminal peptide of the Hb β-chain.

### Red blood cell trapping

Red blood cells were separated from a rabbit blood sample by centrifugation. The red blood cells were dialysed into phosphate buffer (100 mM, pH 7.4) overnight. NaHCO_3_ (6.8 mg) was dissolved in phosphate buffer (1 mL, 50 mM, pH 7.4), added to the red blood cell solution (representing 3.88 mg total red blood cell protein), and the mixture was transferred to the Titration Manager. A freshly made solution of Et_3_OBF_4_ (280 mg, 1.47 mmol) in dH_2_O (1 mL) was added to the mixture in three portions while the pH was maintained by the automated addition of NaOH solution (1 M). The reaction mixture was stirred for 1 h then dialysed against dH_2_O (1 L) overnight. The sample was then centrifuged, an aliquot (100 μL) was taken from the supernatant and digested using trypsin. ESI-MS data confirmed a trapped carbamate on the *N*-terminal peptide of the Hb β-chain.

### *Arabidopsis thaliana* plant growth

Arabidopsis seeds were plated onto 0.8% (w/v) plant agar containing 4.4 g/L Murashige and Skoog salt mixture and incubated at 4 °C for 48 h in the dark. The seeds were then incubated at 22 °C with 12 h of light for one week before planting into jiffy pellet soil plugs (LBS Horticulture) and grown at 22 °C with 12 h of daylight for 5 weeks.

### *Arabidopsis* protein extraction

*Arabidopsis* leaves (5 g dry weight) were ground in a pestle and mortar in the presence of liquid N_2_. Pre-chilled extraction phosphate buffer (4 °C, 100 mM, 15 mL, pH 7.4) was added to the leaves with sand and poly(vinylpolypyrrolidone) (PVPP) and further grinding was performed. The mixture was passed through Miracloth (Millipore) on ice, and the filtrate was centrifuged at 4500*g* for 10 min at 4 °C. The supernatant, containing soluble proteins, was used for trapping experiments.

### *Arabidopsis thaliana* leaf lysate trapping

Extracted protein solution (3 mg, Bradford Assay) was dissolved in phosphate buffer (2 mL, 50 mM, pH 7.4). NaHCO_3_ (1.7 mg, 0.02 mmol) was dissolved in phosphate buffer (1 mL, 50 mM, pH 7.4), added to the protein solution, and the mixture was transferred to the Titration Manager. A freshly made solution of Et_3_OBF_4_ (280 mg, 1.47 mmol) in dH_2_O (1 mL) was added to the mixture in three portions while the pH was maintained by the automated addition of NaOH solution (1 M). The reaction mixture was stirred for 1 h then dialysed against dH_2_O (1 L) overnight. The sample was then centrifuged, an aliquot (100 μL) was taken from the supernatant. This was diluted to 1 μg ml^−1^ and taken forward for trypsin digestion.

### *At*PRX34 recombinant protein expression

*At*PRX34_31-353_ wild type, K262A, K268A, K262Q and K268Q encoding open reading frames were cloned into the *Nde*I and *Bam*HI sites of pET14b by commercial gene synthesis (Genscript). pET14b-*At*PRX34_31-353_ (*At*PRX34_31-353_ wild type, K262A, K268A, K262Q and K268Q mutant proteins) was expressed in *Escherichia coli* BL21(DE3) pLysS at 20 °C for 16 h with 400 μM isopropyl-β-D-thiogalactoside (IPTG). Pelleted bacteria (20 mL) were suspended in sonication buffer (137 mM NaCl, 2.7 mM KCl, 10 mM Na_2_HPO_4_, 1.8 mM KH_2_PO_4_, 5 mM dithiothreitol, 1% (v/v) Triton-X100), lysed by sonication (180 s) and centrifuged (75,500*g*, 30 min, 4 °C). The pellet was suspended in sonication buffer and sonication and pellet wash repeated three times. The pellet was subsequently suspended in denaturation buffer (6 M guanidine hydrochloride, 100 mM Tris HCl pH 8.5, 10 mM EDTA), further sonicated (180 s), and incubated at 4 °C with gentle rocking. Protein was dialysed against refolding buffer (3.25 M urea, 50 mM Tris HCl pH 9.5, 5% (v/v) glycerol, 40 mM CaCl_2_, 0.7 mM oxidised glutathione, 0.21 mM reduced glutathione, 10 µM hemin) at 4 °C for 16 h. Dialysed protein was incubated with Ni^2+^-nitrilotriacetic acid resin (Qiagen) for 1 h. The resin was transferred to a column and allowed to settle while the flow-through was collected. The column was washed with three column volumes of wash buffer (50 mM Tris pH 7.5, 100 mM NaCl, 10 mM Imidazole). The protein was then eluted with a gradient of imidazole in wash buffer (range 50–250 mM) with detection at 280 nm. The eluted fractions were analysed by SDS-PAGE (Supplementary Figure 3).

### *At*PRX34 recombinant protein assay

*At*PRX34_31-353_ wild type or mutant protein was added to a mixture of 50 mM Tris HCl pH 7.5, 1.6 mM 2-methoxyphenol and 1.2 mM hydrogen peroxide. The mixture was incubated at 25 °C for 30 min and activity monitored through the production of 1,2-benzoquinone by absorbance readings taken at 470 nm. CO_2_ minus experiments were performed in a CO_2_-scrubbed atmospheric chamber with reagents pre-equilibrated to remove CO_2_. Biologically independent experiments were performed on independently made protein preparations.

### Mass spectrometry and data handling

Following the trapping reaction proteins were either digested using the filter aided sample preparation method (FASP) or using gel-aided sample preparation (GASP) as described without modifications^34,35^. The resulting peptide solution was desalted with home packed C18 stage tips^36^. The resulting peptide mixture was dried down and dissolved in 15 μL 4% acetonitrile (MeCN), 0.05% trifluoroacetic acid (TFA). 20% of this was analysed by LCMSMS carried out either on a Qstar Pulsar *i* QqTOF mass spectrometer (Sciex) coupled to an Ultimate 3000 nano-HPLC instrument, an LTQ Orbitrap XL mass spectrometer (Thermo) also coupled to an Ultimate 3000 nano-HPLC instrument or a TripleTOF 6600 QqTOF mass spectrometer (Sciex) coupled to an Eksigent ekspert nanoLC 425 instrument. Peptides were concentrated on a Pepmap C18 trap column (300 μm ID × 5 mm) and separated on a Pepmap C18 reversed phase analytical column (Dionex, UK) (3 μm particles, 75 μm ID × 250 mm). Separations were carried out at 300 nL min^−1^ on a linear gradient from 96% A (0.05% formic acid), 4% B (0.05% formic acid, 80% acetonitrile) to 35% B over 70 min followed by a second linear gradient from 35% B to 65% B over 7 min. Peptides eluted from the LC gradient were injected online to the mass spectrometers using the following parameters: QStar (IDA mode, mass range 300–1600 Da, MS accumulation time 1 s, ion source voltage 2300 V, 3 MSMS spectra per cycle, MSMS mass range 100–1600 Da, MSMS accumulation time 3 s), Oritrap LTQ XL (lock mass enabled, mass range 400–1800 Da, resolution 60,000 at 400 Da, 10 MSMS spectra per cycle, collision induced dissociation (CID) at 35% normalised CE, rejection of singly charged ions), TripleTOF 6600 (IDA mode, mass range 400–1250 Da, MS accumulation time 250 ms, ion source voltage 2400 V, 30 MSMS spectra per cycle, MSMS mass range 100–1500 Da, MSMS accumulation time 50 ms).

Raw data (.raw and .wiff) were converted to *.mgf peak lists using MSconvert^37^. Peak lists were then searched using X!Tandem^30^ against a concatenated database consisting of the *A. thaliana* proteome (Uniprot entry version 139 updated 07/07/2017), cRAP (version 01/01/2012)^38^ and the reverse fasta files for both^30^. The following search parameters were used: Parent mass error, 20 ppm. Fragment mass error, 30 ppm (0.4 Da for QStar and Orbitrap XL data). Missed cleavage sites allowed, (1) Fixed modification of + 57.0215@C. Oxidation of methionine was allowed for as a variable modification. Three rounds of refinement were carried out to detect further modified peptides permitting additional variable modifications in round 1 (0.98401@N, 79.9663@S, 79.9663@T, 79.9663@Y, 15.999@M, 15.999@W, 0.98401@Q, 14.016@C, 14.016@D, 14.016@E, 14.016@H, 28.0313@K, 28.0313@E, 28.0313@D), in round 2 (14.016@D, 31.9898@M, 31.9898@W, -18.011@S, -18.011@T, -57.021@C, 14.016@Q, 57.021@H, 57.021@D, 57.021@E, 72.0211@K) and in round 3 (56.0626@K). The mass spectrometry proteomics data have been deposited to the ProteomeXchange Consortium (http://proteomecentral.proteomexchange.org) via the PRIDE partner repository^39^ with the data set identifier PXD007753.

### Statistical analysis

Data was analysed by two-tailed *t*-test or one-way ANOVA as indicated in the text. Normal distribution of the data for ANOVA of Fig. 8 was confirmed by the Shapiro–Wilk test. Homogeneity of variances was rejected for all factors examined using the Levene Test. Data were therefore examined using a non-parametric Kruskall–Wallis test with Dunn Bonferroni post-hoc test for pairwise comparisons with unequal variances.

### Data availability

The mass spectrometry proteomics data have been deposited to the ProteomeXchange Consortium (http://proteomecentral.proteomexchange.org) via the PRIDE partner repository^38^ with the data set identifier PXD007753. The datasets generated during the study are available from the corresponding author on reasonable request.

## Electronic supplementary material

Supplementary Information

Description of Additional Supplementary Files

Supplementary Data 1
