# Author Correction: The identification of carbon dioxide mediated protein post-translational modifications

**DOI:** 10.1038/s41467-018-06711-2

**Published:** 2018-10-03

**Authors:** Victoria L. Linthwaite, Joanna M. Janus, Adrian P. Brown, David Wong-Pascua, AnnMarie C. O’Donoghue, Andrew Porter, Achim Treumann, David R. W. Hodgson, Martin J. Cann

**Affiliations:** 1 Department of Biosciences, Durham University, South Road, Durham, DH1 3LE UK; 2 Biophysical Sciences Institute, Durham University, South Road, Durham, DH1 3LE UK; 3 Department of Chemistry, Durham University, South Road, Durham, DH1 3LE UK; 4 Centre for Sustainable Chemical Processes, Durham University, South Road, Durham, DH1 3LE UK; 5 NUPPA, The Protein Facility, Newcastle University, Cookson Building, Newcastle upon Tyne, NE2 4HH UK

Correction to: *Nature Communications* ; 10.1038/s41467-018-05475-z; published online 6 August 2018

The original version of this Article omitted the following from the Acknowledgements:

‘This work was support by EPSRC grant EP/K504336/1 and Leverhulme Trust grant RPG-2016-017.’

This has been corrected in both the PDF and HTML versions of the Article.

