# Supplementary material for: The identification of carbon dioxide mediated protein post-translational modifications
Source: Nat Commun. 2018 Aug 6;9:3092. doi: 10.1038/s41467-018-05475-z (PMC6078960; doi:10.1038/s41467-018-05475-z)
Supplement: Supplementary file 1 — Supplementary Information [file 41467_2018_5475_MOESM1_ESM.pdf]

SUPPLEMENTARY INFORMATION

The identification of carbon dioxide mediated protein post-translational modifications

Linthwaite *et al.*

Supplementary Figures

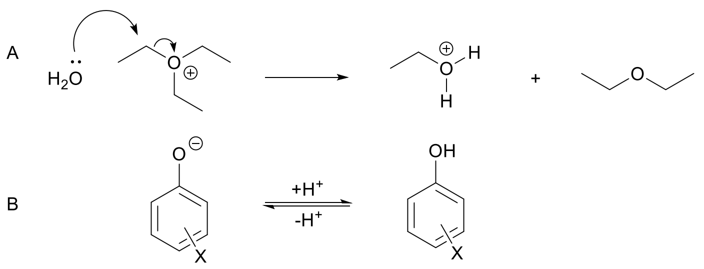

Supplementary Figure 1. Monitoring triethyloxonium ion hydrolysis spectrophotometrically. A. Formation of acid upon hydrolysis of triethyloxonium ion. **B.** Proton transfer in phenol indicators.

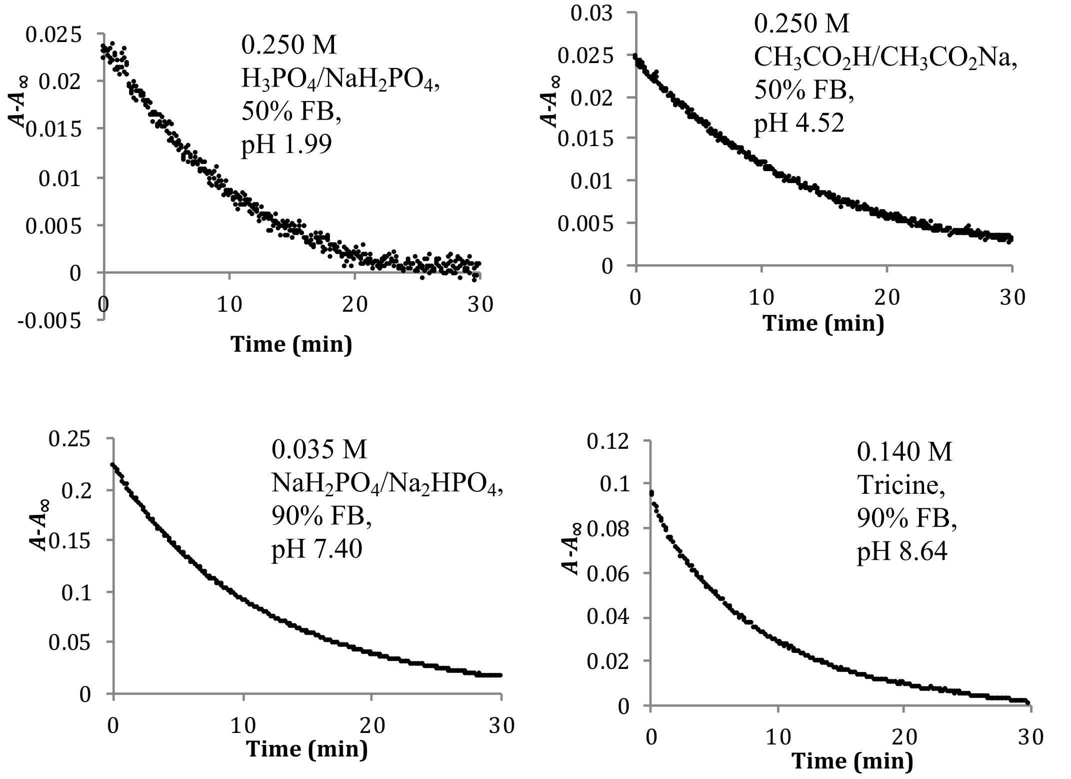

Supplementary Figure 2. Representative raw data for the hydrolysis of triethyloxonium tetrafluoroborate (15 mM) in aqueous buffered solutions. I = 1.00 M ($NaBF_4$) at 25 °C. 2-Aminobenzoic acid (0.5 mM, λ_{max} = 328 nm), 2,4-dinitrophenol (0.18 mM, λ_{max} = 401 nm), *p*-nitrophenol (0.27 mM, λ_{max} = 398.5 nm) and 3-nitrophenol (0.84 mM, λ_{max} = 410 nm) were used as indicators at pH 1.99, 4.52, 7.40 and 8.64, respectively.

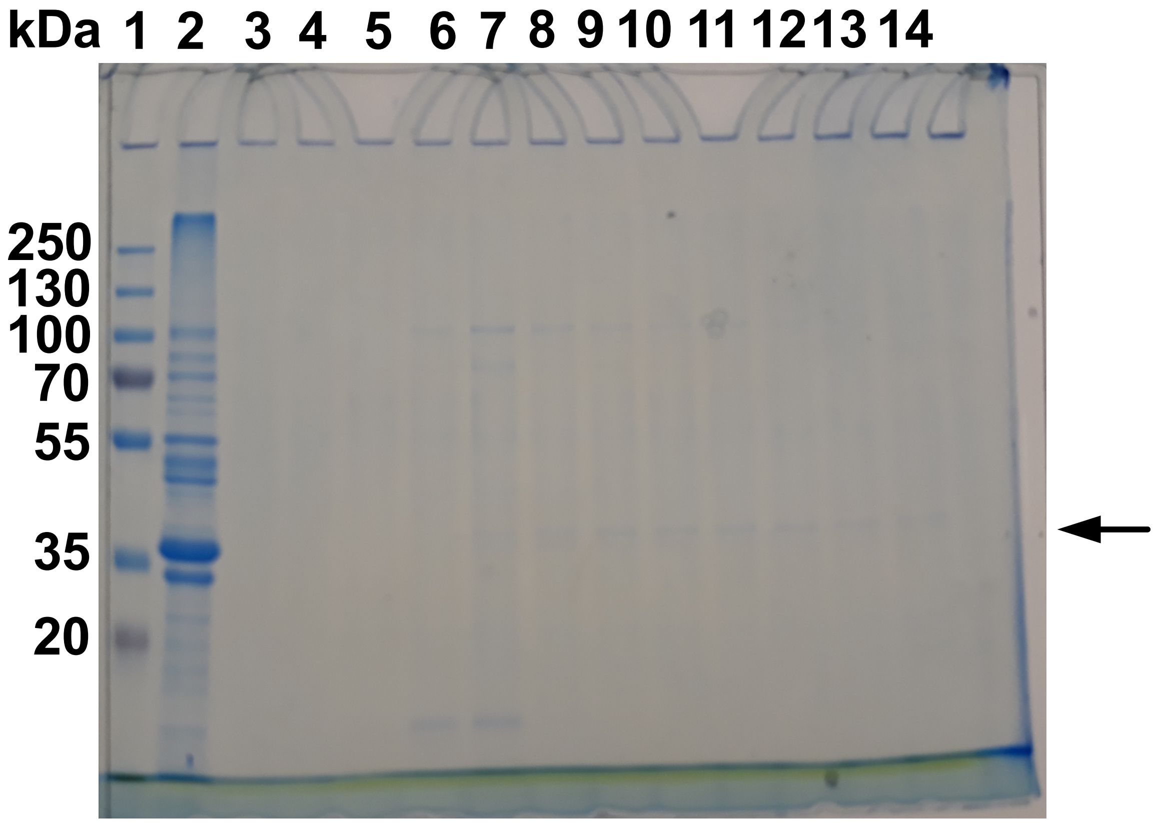

Supplementary Figure 3. Recombinant *At*PRX34$_{31\text{-}353}$ purification. 1. ~10 µg total *At*PRX34$_{31\text{-}353}$ after dialysis against refolding buffer. Lanes 2-14 are 10 µL fractions of 1 mL elutions at 25 mM (2), 50 mM (3), 100 mM (4), 125 mM (5), 150 mM (6), 160 mM (8), 170 mM (9), 180 mM (10), 190 mM (11), 200 mM (12), 225 mM (13) and 250 mM (14) imidazole. kDa indicates molecular weight markers. Arrow indicates position of *At*PRX34$_{31\text{-}353}$.

Supplementary Tables

Supplementary Table 1. Components of reaction mixtures employed for studying pH dependence of triethyloxonium tetrafluoroborate (15 mM) hydrolysis.

Buffer	Initial pH	Indicator	λ_{max} (nm)
0.1-1.0 M HCO_2H/HCO_2K 10% FB	2.57	0.18 mM 2,4 dinitrophenol	401
0.1-1.0 M HCO_2H/HCO_2K 90% FB	4.67	0.18 mM 2,4 dinitrophenol	401
0.05-0.5 M KH_2PO_4/K_2HPO_4 50% FB	6.67	0.27 mM *p*-nitrophenol	398.5
0.035-0.35 M KH_2PO_4/K_2HPO_4 90% FB	7.72	0.27 mM *p*-nitrophenol	398.5
0.025-0.25 M H_3PO_4/NaH_2PO_4 50% FB	1.99	0.5 mM 2-aminobenzoic acid	328
0.025-0.25 M H_3PO_4/NaH_2PO_4 90% FB	3.01	0.5 mM 2-aminobenzoic acid	328
0.025-0.25 M CH_3CO_2H/CH_3CO_2Na 50% FB	4.52	0.18 mM 2,4 dinitrophenol	401
0.025-0.25 M CH_3CO_2H/CH_3CO_2Na 90% FB	5.20	0.18 mM 2,4 dinitrophenol	401
0.05-0.50 M NaH_2PO_4/Na_2HPO_4 50% FB	6.61	0.27 mM *p*-nitrophenol	398.5
0.035-0.35 M NaH_2PO_4/Na_2HPO_4 90% FB	7.40	0.27 mM *p*-nitrophenol	398.5
0.02-0.2 M Tricine/Tricine$^-$ 90% FB	8.64	0.84 mM 3-nitrophenol	410

Supplementary Table 2. Observed rate constants for the hydrolysis of triethyloxonium tetrafluoroborate.

Buffer	pH	k_{obs} (s^{-1})	log (k_{obs})
0.1-1.0 M HCO_2H/HCO_2K 10% FB	2.57	1.56×10^{-3}	-2.81
0.1-1.0 M HCO_2H/HCO_2K 90% FB	4.67	1.46×10^{-3}	-2.84
0.05-0.5 M KH_2PO_4/K_2HPO_4 50% FB	6.67	2.55×10^{-3}	-2.59
0.035-0.35 M KH_2PO_4/K_2HPO_4 90% FB	7.72	2.71×10^{-3}	-2.57
0.025-0.25 M H_3PO_4/NaH_2PO_4 50% FB	1.99	1.29×10^{-3}	-2.89
0.025-0.25 M H_3PO_4/NaH_2PO_4 90% FB	3.01	1.32×10^{-3}	-2.88
0.025-0.25 M CH_3CO_2H/CH_3CO_2Na 50% FB	4.52	1.32×10^{-3}	-2.88
0.025-0.25 M CH_3CO_2H/CH_3CO_2Na 90% FB	5.20	1.55×10^{-3}	-2.81
0.05-0.50 M NaH_2PO_4/Na_2HPO_4 50% FB	6.61	3.37×10^{-3}	-2.47
0.035-0.35 M NaH_2PO_4/Na_2HPO_4 90% FB	7.40	5.52×10^{-3}	-2.26
0.02-0.2 M Tricine/Tricine$^-$ 90% FB	8.64	1.89×10^{-3}	-2.72

Supplementary Methods

Determination of Pseudo-First Order Hydrolysis Rate Constants by UV-Vis Spectrophotometry

The extent of hydrolysis of triethyloxonium ion was measured spectrophotometrically (Supplementary Figure 1) using a technique adapted from a procedure to monitor hydrolysis of phosphorus chlorides [1] and carbon dioxide hydration [2]. As triethyloxonium is hydrolysed, acid is generated stoichiometrically (Supplementary Figure 1A). The formation of acid is measured spectrophotometrically by employing a substituted phenol (Supplementary Figure 1B) indicator possessing a pK_a value within 0.5 pH units of the buffer solution.

Proton transfer at the phenolic indicator compounds results in a change in absorbance. This coupled system permits monitoring of the hydrolysis of triethyloxonium ion by UV-Vis spectrophotometry as proton transfer within the phenolate system is diffusion controlled and hence substantially faster than triethyloxonium hydrolysis. The indicator and buffer combinations used for the hydrolysis studies are shown in Supplementary Table 1, which also includes the initial measured pH and λ_{max} values. Aqueous buffer solutions had an ionic strength, I = 1.00 M ($NaBF_4$ or KCl).

Representative spectral data acquired using a CARY100 UV-Visible spectrophotometer for several buffer systems are shown in Supplementary Figure 2. Data for all buffer systems showed exponential decays in absorbance, while absolute changes in absorbance over the time scale of reaction tended to be larger at lower buffer concentrations. Non-linear least squares fitting of A-A_∞ values over time to a first order exponential decay curve yielded values for the pseudo first order rate constants for hydrolysis (k_{obs}, Supplementary Table 2).

Stock solutions of triethyloxonium tetrafluoroborate were prepared immediately before use in dry acetonitrile solution and stored at 0 °C. For UV-Vis spectrophotometer experiments buffer solutions were prepared in cuvettes by mixing stock solutions of buffer and indicator and allowed to equilibrate to 25 °C in the cell holder. Hydrolysis of triethyloxonium was initiated by addition of stock solutions of triethyloxonium tetrafluoroborate to equilibrated buffer-indicator solutions. The volume of this stock solution was equivalent to 1 % of the final total reaction mixture volume. Reactions were followed for at least six half-lives and performed in triplicate at four different buffer concentrations to assess for buffer catalysis.

Absorbance measurements were obtained using a Cary 100 UV-visible spectrophotometer and Applied Photophysics SX-17MV stopped flow apparatus. For measurements on the Cary 100, a Peltier heating/refrigeration unit were employed to maintain a constant temperature of 25 °C and measurements were taken in 1 ml quartz cuvettes with a 1 cm path length. The λ_{max} of each indicator and buffer system was determined in the absence of triethyloxonium from spectra acquired from the Scan program. The Kinetics program was used to monitor changes at this λ_{max} after addition of triethyloxonium tetrafluoroborate solution. KaleidaGraph software was used to fit processed data (A-A_{∞}) and acquire rate constants.

Measurements of pH of aqueous buffers were determined at 25 °C using a MeterLab™ PHM 290 pH-Stat Controller equipped with a radiometer combination electrode filled with saturated KCl solution. Calibration was performed with calibration buffers pH 4, pH 7 and pH 10.0.

Supplementary References

1 Delley, R. J., O'Donoghue, A. C. & Hodgson, D. R. Hydrolysis studies of phosphodichloridate and thiophosphodichloridate ions. *J Org Chem* **77**, 5829-5831, doi:10.1021/jo300808m (2012).

2 Khalifah, R. G. The carbon dioxide hydration activity of carbonic anhydrase. I. Stop-flow kinetic studies on the native human isoenzymes B and C. *J Biol Chem* **246**, 2561-2573 (1971).
