# Supplementary material for: The identification of carbon dioxide mediated protein post-translational modifications
Source: Nat Commun. 2018 Aug 6;9:3092. doi: 10.1038/s41467-018-05475-z (PMC6078960; doi:10.1038/s41467-018-05475-z)
Supplement: Supplementary file 2 — Description of Additional Supplementary Files [file 41467_2018_5475_MOESM2_ESM.pdf]

Description of Additional Supplementary Files

File Name: Supplementary Data 1
Description: **The identification of CO_2-binding proteins.** High resolution MSMS spectra data for peptides that were identified with ethyl-trapped carbamates on Lys residues.
